# Modeling MEN1 with Patient-Origin iPSCs Reveals GLP-1R Mediated Hypersecretion of Insulin

**DOI:** 10.3390/cells11152387

**Published:** 2022-08-03

**Authors:** Ziqi Cheng, Dongsheng Guo, Aynisahan Ruzi, Tingcai Pan, Kai You, Yan Chen, Xinping Huang, Jiaye Zhang, Fan Yang, Lizhi Niu, Kecheng Xu, Yin-Xiong Li

**Affiliations:** 1 Center for Health Research, Guangzhou Institutes of Biomedicine and Health, Chinese Academy of Sciences, Guangzhou 510530, China; cheng_ziqi@gibh.ac.cn (Z.C.); dongsheng.guo@umassmed.edu (D.G.); aynisa0512@163.com (A.R.); lingshan@163.com (T.P.); shengminguk@163.com (K.Y.); chen_yan@gibh.ac.cn (Y.C.); huang_xinping@gibh.ac.cn (X.H.); nonsenes@sina.com (J.Z.); 2 University of Chinese Academy of Sciences, Beijing 100049, China; 3 Key Laboratory of Stem Cell and Regenerative Medicine, Guangzhou Institutes of Biomedicine and Health, Chinese Academy of Sciences, Guangzhou 510530, China; 4 CAS Key Laboratory of Regenerative Biology, Guangzhou Institutes of Biomedicine and Health, Chinese Academy of Sciences, Guangzhou 510530, China; 5 Guangdong Provincial Key Laboratory of Biocomputing, Guangzhou Institutes of Biomedicine and Health, Chinese Academy of Sciences, Guangzhou 510530, China; 6 Ministry of Education CNS Regeneration Collaborative Joint Laboratory, Guangdong-Hongkong-Macau Institute of CNS Regeneration, Jinan University, Guangzhou 510632, China; yeungkailam905@gmail.com; 7 Guangzhou Fuda Cancer Hospital, Guangzhou 510305, China; niuboshi@fudahospital.com (L.N.); xukc@vip.163.com (K.X.); 8 State Key Laboratory of Respiratory Disease, Guangzhou 510000, China; 9 China-New Zealand Joint Laboratory of Biomedicine and Health, Guangzhou 510530, China

**Keywords:** disease modeling, MEN1, GLP-1R, hyperinsulinemia, β-cell differentiation

## Abstract

Multiple endocrine neoplasia type 1 (MEN1) is an inherited disease caused by mutations in the *MEN1* gene encoding a nuclear protein menin. Among those different endocrine tumors of MEN1, the pancreatic neuroendocrine tumors (PNETs) are life-threatening and frequently implicated. Since there are uncertainties in genotype and phenotype relationship and there are species differences between humans and mice, it is worth it to replenish the mice model with human cell resources. Here, we tested whether the patient-origin induced pluripotent stem cell (iPSC) lines could phenocopy some defects of MEN1. In vitro β-cell differentiation revealed that the percentage of insulin-positive cells and insulin secretion were increased by at least two-fold in MEN1-iPSC derived cells, which was mainly resulted from significantly higher proliferative activities in the pancreatic progenitor stage (Day 7–13). This scenario was paralleled with increased expressions of prohormone convertase1/3 (PC1/3), glucagon-like peptide-1 (GLP-1), GLP-1R, and factors in the phosphatidylinositol 3-kinase (PI3K)/AKT signal pathway, and the GLP-1R was mainly expressed in β-like cells. Blockages of either GLP-1R or PI3K significantly reduced the percentages of insulin-positive cells and hypersecretion of insulin in MEN1-derived cells. Furthermore, in transplantation of different stages of MEN1-derived cells into immune-deficient mice, only those β-like cells produced tumors that mimicked the features of the PNETs from the original patient. To the best of our knowledge, this was the first case using patient-origin iPSCs modeling most phenotypes of MEN1, and the results suggested that GLP-1R may be a potential therapeutic target for MEN1-related hyperinsulinemia.

## 1. Introduction

MEN1 is a rare and dominantly inherited familial cancer syndrome. This disease is characterized by an increased incidence of endocrine tumors in target tissues (mainly parathyroid, pancreatic islet, and anterior pituitary) [1]. MEN1 patients suffer from hormonal abnormalities leading to body malfunction with a high mortality rate if left untreated. Approximately 40% of those *MEN1* mutations carriers develop PNETs, and 10% of those patients have insulinoma [2]. Patients with insulinoma of MEN1 often experience hypoglycemia-induced syncope, which affects their daily life and could be life-threatening [3].

The *MEN1* gene encodes a 68 KD nuclear protein, menin, which interacts with various factors for multiple functions, including gene transcriptional regulation, chromosome integrity maintenance, and DNA replication [4,5,6]. There are currently over 1336 different mutations reported in MEN1, and there are no obvious mutational hotspots, with the majority of mutations (>70%) resulting in truncation and dysfunction of menin [2,7,8]. The multiple functions of menin and the diversity of *MEN1* mutations make it very difficult to establish a mice disease model for studying all of the detailed pathogenesis of MEN1.

The proliferation of the pancreatic β-cells is decreased after birth [9], however, its cell cycle can be accelerated in situations of increased metabolic demand, including obesity and pregnancy and menin also contributes to this process [10]. Menin preferentially represses the proliferation of islet cells, mainly β-cells [11,12]. Menin promotes histone methylation and the expression of p27 and p18 to inhibit the proliferation of β-cells [13,14,15]. Menin also regulates β-cell mitogens by switching on the PI3K/AKT pathway and regulating its substrates, such as forkhead box protein O 1(FOXO1) and cAMP responsive element binding protein (CREB) [16,17,18]. However, how menin regulates β-cell proliferation is not well-understood.

It was reported that menin regulated endocrine cell proliferation by controlling the secretion of hormones in the islet microenvironment [19,20], but the exact mechanism is not clear. GLP-1 is a peptide hormone that is mainly secreted by intestinal L-cells or pancreatic α-cells. Both GLP-1 and glucagon are generated from proglucagon with different cleavages by PC1/3 or PC2, respectively [21].

While there is a shortage of β-cells, α-cells would secrete GLP-1 to stimulate glucose-dependent insulin production by binding its cell surface receptor, GLP-1R [22,23,24]. Menin suppresses GLP-1R transcription and protein kinase A (PKA)-mediated phosphorylation of FOXO1 and CREB to downregulate β-cell proliferation and insulin secretion [25]. PKA, the downstream of GLP-1R, leads Ser487 phosphorylation of menin, reducing its binding to the insulin promoter and relieving its inhibitory effect on insulin secretion [26]. A study revealed that an α-cell specific mutation of *Men1* resulted in the increased self-proliferation and the trans-differentiation of α-cells into insulin-positive cells, which subsequently developed into insulinoma [27]. Therefore, whether menin controls β-cell proliferation by regulating the secretion of GLP-1 is unknown.

Menin is highly conserved among species and the majority of missense mutations (68%) occur in highly conserved sequences in MEN1 patients, suggesting the feasibility of animal models to investigate MEN1 disease [28]. However, the disease model of PNETs in MEN1 based on gene-edited mice appears to be less reliable. First, the type and the probability of a developed tumor in the mouse model were different from those in humans, with a 40% probability of PNETs development in MEN1 patients but 79.2% in *Men1*-mutant rodents [29]. Second, there are differences between the mouse and the human pancreas; structurally, human islets are homogeneous in the pancreas, whereas mouse islets are concentrated in the central of the pancreas [30]; molecularly, mouse β-cells do not express specific markers such as MAFB and GLUT2, which are unique to human β-cells and important for pancreatic endocrine identity. Third, only 10% of the pancreas is required to maintain glucose homeostasis in rodents, but in humans, a lack of 50% of the pancreas can lead to development of insulin-dependent diabetes [31,32]. Fourth, the proliferative capacity of rodent β-cells under stress is much greater than that of human β-cells, but the exact mechanism is unknown [33]. Therefore, a MEN1 disease model based on human cells would be more reliable in understanding the specific pathogenesis of MEN1 in humans, especially for PNETs.

Here, we established a patient iPSCs-based modeling of MEN1 disease by differentiating MEN1-iPSCs into insulin-producing cells and identified that the overactivation of GLP-1R and PI3K/AKT signaling were the key events of MEN1 pathology.

## 2. Materials and Methods

### 2.1. Cell Culture

The methods of urine cells collection and iPSCs reprogramming were previously reported [34,35]. MEN1-patient-specific mother iPSCs (MOTHER-iPSC) were generated from urine cells of a 59-year-old female patient with MEN1 syndrome [36]. MEN1-patient-specific son iPSCs (SON-iPSC) were generated from urine cells of a 23-year-old male patient with MEN1 syndrome [37]. WT iPSCs (WT-iPSC) were generated from urine cells of a healthy person [38]. All iPSCs were cultured at 37 °C under 5% CO_2_ in air with mTeSR1 (STEM-CELL Technologies, Vancouver, BC, Canada) in 1:100 Matrigel (Corning, Manassas, VA, USA)-coated dishes. Cells were passaged at 1:4 with accutase (Sigma, St Louis, MO, USA) and cultured for the first 24 h in mTeSR1with 5μM Y-276342 (Selleck, Munich, Germany) and then we replaced the medium with mTeSR1 without Y-276342.

### 2.2. Cell Differentiation

Published protocol was used to differentiate stem cells into insulin-producing cells [39]. In short, stem cells were plated into Matrigel-coated 24-well plates at a density of 10 × 10^4^ cells per well and cultured 3 days. When the cell density reached about 70% confluence, we started the first stage induction of definitive endoderm cells (DECs). The cells were incubated with RPMI 1640 (Gibco, Carlsbad, CA, USA) medium with B27 minus insulin (Gibco), 100 ng/mL activin A (PeproTech, Rotterdam, The Netherlands), and 3 µM CHIR99021 (Selleck) for 24 h, and then replaced by RPMI 1640 medium with B27 minus insulin and 100 ng/mL activin A for another 48 h. The second stage of differentiation was a 7-day induction of pancreatic progenitor cells (PPCs), the medium and compounds were DMEM/F12 with B27, 2 μM retinoic acid (Sigma), 10 µM SB431542 (Selleck), and 1 µM dorsomorphin (Selleck), and the culture medium was changed every 48 h. The third stage was insulin-producing cell (IPCs) differentiation, and the condition was DMEM/F12 (HyClone, Logan, UT, USA) medium with B27, 10 µM dexamethasone (Enzo Life Sciences, Farmingdale, NY, USA), 10 µM forskolin (Selleck), 5 µM Repsox (Selleck), and 10 mM nicotinamide (STEMCELL Technologies) for 12 days. The GLP-1 and PI3K signaling were intervened at the beginning of IPC stage (Day 11) by administration of GLP-1 agonist, antagonist or PI3K inhibitor (50 ng/mL Exendin-4 (TOCRIS, Minneapolis, MN, USA), 50 ng/mL Exendin-3 (9-39) (Chem Cruz, Santa Cruz, Dallas, TX, USA), or 3 μM LY-294002 (Selleck)).

### 2.3. Immunofluorescence Microscopy

**Figure 1:**
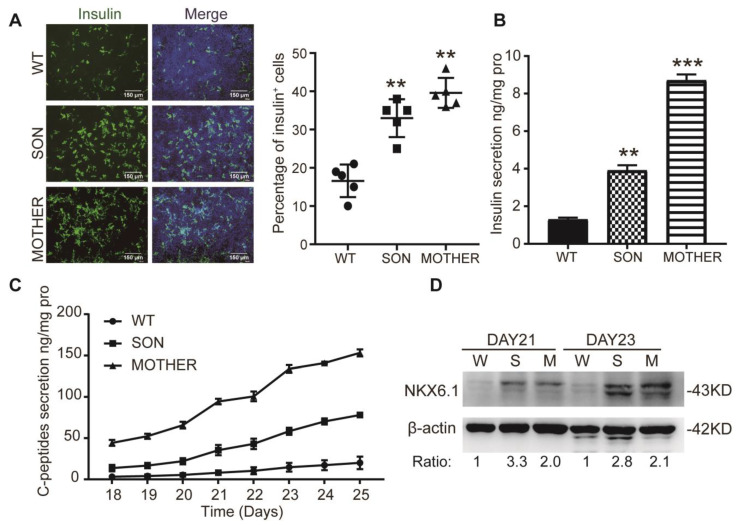
Significant increased percentage of insulin-positive cells and insulin production in MEN1-IPCs. (**A**) Insulin expression in IPC stage, *n* = 5, scale bar: 150μm. (**B**) ELISA measurements of secreted insulin in IPC stage, *n* = 3. (**C**) The secretion of C-peptides during the differentiation process, *n* = 3. (**D**) The expression of NKX6.1 in IPC stage with the number below representing the fold change after being normalized, *n* = 3. W-WT, S-SON, M-MOTHER. The bar plots (*n* ≥ 3 wells per group) are mean ± SEM. ** *p* < 0.01, *** *p* < 0.001.

**Figure 2:**
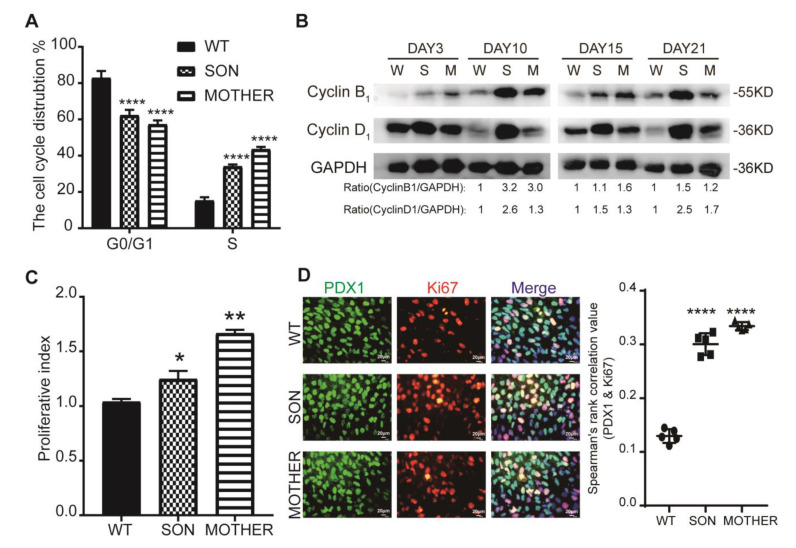
Increased insulin production in MEN1-IPCs was mainly linked with the proliferative abilities. (**A**) Cell cycle analysis by PI via flow cytometry in PPC stage, *n* = 3. (**B**) Cell cycle proteins, Cyclin D_1_ and Cyclin B_1_ were analyzed during the differentiation process from PPC stage to IPC stage, respectively, with the number below representing the fold change after being normalized *n* = 3. (**C**) Proliferative analysis by CFSE via flow cytometry since Day 7–13, *n* = 3. (**D**) The proliferation of PPC stage was identified by Ki67 and PDX1 with the doubled staining and quantitative information, and the correlation was calculated by image J, *n* = 5, scale bar: 150μm. W-WT, S-SON, M-MOTHER. The bar plots (*n* ≥ 3 wells per group) are mean ± SEM. * *p* < 0.05, ** *p* < 0.01, and **** *p* < 0.0001.

**Figure 3:**
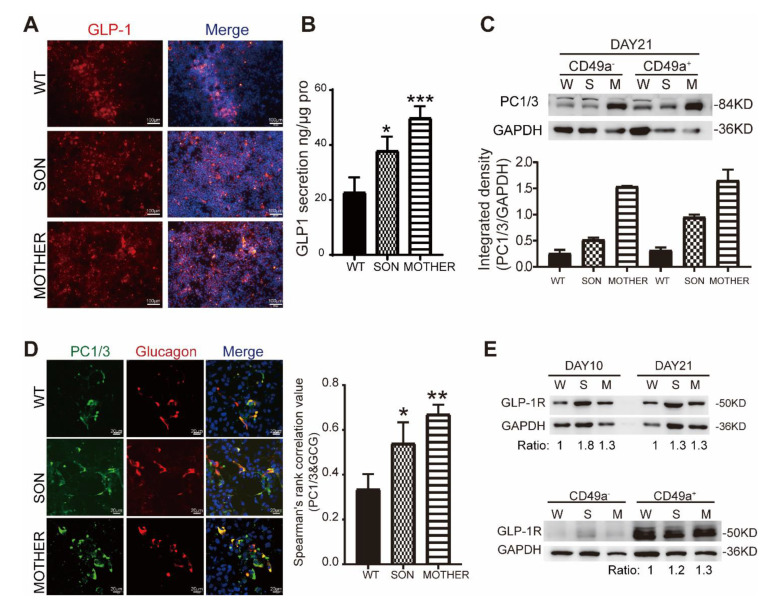
The secretion of GLP-1 rose in MEN1-derived cells with increased expression of GLP-1R depended on the high percentage of β-like cells. (**A**) The immunostaining of GLP-1 in IPC stage scale bar: 100 μm. (**B**) ELISA measurement of GLP-1 secretion in IPC stage, *n* = 3. (**C**) The protein expression of PC1/3 in CD49a^+^ and CD49a^−^ IPCs, *n* = 5. (**D**) The immunostaining of PC1/3 and proglucagon in IPC stage and the correlative calculation by image J, *n* = 5, scale bar: 20 μm. (**E**) The expression of GLP-1R in PPC and IPC stage and after cell-sorting with CD49a; the number below is representing the fold change after being normalized, *n* = 3; W-WT, S-SON, M-MOTHER. The bar plots (*n* ≥ 3 wells per group) are the mean ± SEM. * *p* < 0.05, ** *p* < 0.01, and *** *p* < 0.001.

The harvested cells were fixed in 4% paraformaldehyde (PFA) for half an hour at room temperature. Cells were then washed with PBS 3 times. Next, cells were blocked and permeabilized with PBS containing 0.2% Triton X-100 and 10% FBS for 2 h at room temperature and washed with PBS 3 times. Cells were incubated with primary antibodies diluted in PBS containing 10% FBS at 4 °C overnight. The next day, cells were washed 3 times with PBS and incubated with appropriate secondary antibodies at 1:1000 dilutions for 1 h at room temperature. Cells were washed 3 times with PBS before being stained with DAPI (Sigma) for 15 min. We imaged the sample with a microscope (OLYMPUS IX73, Tokyo, Japan). All antibodies are listed in Table S1 and dilutions of antibodies used in the immunofluorescence microscopy were performed according to the suppliers’ instructions. The fluorescence intensity was calculated by Image J (NIMH; Bethesda, MD, USA) for quantitative analysis, and the representative images were chosen from 5 independent experiments performed in total and are shown in Figures 1–3,5 and Figures S1–S3.

### 2.4. Cell Cycle and Cell Proliferation Assay

Cell cycle was measured by using propidium iodide (PI) (BD, Franklin Lakes, NJ, USA) staining dye. In brief, we collected 5 × 10^5^ cells from target stages and washed them twice with precooled PBS. Cells were fixed with 70% ethanol at 4 °C overnight. After washing with PBS, collected cells were incubated in PBS supplemented with 50μg/mL PI, 100 μg/mL RNase A (Sigma Aldrich, St. Louis, MO, USA), and 0.2% Triton X-100 diluted in PBS at 4 °C for 30 min. Cells were washed twice with PBS before FACS analysis. We used a cell division tracking dye carboxyfluorescein diacetate succinimidyl ester (CFSE) (DOJINDO, Rockville, MD, USA) to measure cell proliferation. A total of 5 × 10^5^ cells were collected and stained with the CFSE dye at 5 μM. The cells were incubated at 37 °C for 10 min, and then washed with RPMI medium twice and cells were stained. We continued to culture the stained cells in a light-proof place for 5 days and collected the cells for FACS analysis. Unstimulated CFSE-labeled cells served as a nondividing control. Generally, 4–5 × 10^4^ cells were counted, and the results were analyzed by ModFit. The representative data were chosen from 3 independent experiments performed in total and are shown in Figure 2 and Figure S3.

### 2.5. Insulin Secretion

To measure insulin secretion, IPCs (Day 21) were sampled. Clusters were washed with Krebs-Ringer bicarbonate HEPES buffer (KRBH; 116 mM NaCl, 4.7 mM KCl, 2.5 mM CaCl_2_ 1.2 mM KH_2_PO_4_, 1.2 mM MgSO_4_, 24 mM HEPES, 25 mM NaHCO_3_, and 0.1% BSA) and were then preincubated in KRBH buffer for 1 h. The cells were then incubated with KRBH buffer containing 2.5 mM glucose at 37 °C for 60 min. The respective conditioned supernatant was collected and analyzed. Clusters were lysed with RIPA lysis buffer, and protein content was measured with a BCA Protein Assay Kit (Thermo Fisher, Waltham, MA, USA). The collected supernatant was used for insulin analysis with a human insulin ELISA kit (STELLUX, Alpco, Salem, NH, USA). The amount of insulin was normalized to the amount of total protein in the corresponding cell lysate. The representative data were chosen from 5 independent experiments performed in total and are shown in Figures 1 and 5.

### 2.6. Animal Model and Cell Transplantation

Immune-deficient NOD-SCID^IL2RG−/−^ mice (NSI mice, GIBH) were used as recipients of differentiated cells. These mice have been shown to support the growth of many human tumor samples [40]. An amount of 1 × 10^6^ differentiated-cells mixed with Matrigel 1:1 were subcutaneous or subrenal capsular transplanted into the 8-week-old NSI mice. All animal experiments were approved by the Animal Welfare Committee of GIBH. All relative protocols were approved by the institutional animal care committee (IACUC No. 2012036).

### 2.7. Histological Analysis

Mice were sacrificed and the tissue were fixed in 4% paraformaldehyde and embedded in paraffin. 5 μm thick tissue sections were cut, prepared, and adhered to coated slides. Hematoxylin and eosin staining was performed according to a standard procedure (Beyotime, Shanghai, China).

### 2.8. Immunohistochemistry

Tumor tissues were fixed with 4% paraformaldehyde (PFA) for 24 h and embedded in paraffin blocks. 5 μm thick tissue sections were prepared and adhered to coated slides. Next, paraffin sections were dewaxed with xylene and graded alcohol and antigen retrieval was performed with antigen retrieval buffer (Tris/EDTA pH 9.0) at high fire in microwave oven for 15 min. Endogenous peroxidase activity was quenched by incubating section in 3% sodium hydroxide in distilled water for 15 min at RT followed by incubating sections in 10% FBS for nonspecific binding at RT for 1 h. All primary antibodies (listed in supplementary Table S1) were incubated for 2 h at RT, washed thrice with PBS for 5 min at RT, and signals were amplified by secondary antibodies (listed in supplementary Table S1). Expression was visualized using DAB (Zsbio, Beijing, China) reagents, counterstained with hematoxylin or DAPI (1:2000), mounted with mounting medium (Vector Labs, Newark, CA, USA), and examined with microscope (Leica DM4B, Wetzlar, Germany).

### 2.9. Western Blot Analysis

Western blot analysis was followed with the routine protocol in our lab [41]. The densities of positive bands were quantified with the Image J.

We performed at least three independent experiments from cell differentiation to Western blot analysis, and all of those independent experiments revealed the same changed trends of tested target proteins, but hardly generated a significant *p*-value caused by the current protocol limitations (the efficiency of β-like cell differentiation and the long 3-week differentiation time). Therefore, we chose protein samples from one batch of cell differentiation to perform three independent Western blots for technical triplicate, and we showed means of the changed fold from three Western blot analyses in related figures.

### 2.10. Statistical Analysis

The data were analyzed with GraphPad Prism 7. Statistical significance was assessed with one-way ANOVA. Data were represented as means ± SEM. The *p*-values, wherever applicable, were calculated based on the means of the experimental replicates. Statistical significance is denoted by * *p* < 0.05, ** *p* < 0.01, *** *p* < 0.001, and **** *p* < 0.0001.

### 2.11. Study Approval

Under the consent of the Ethics Committees of Guangzhou Institutes of Biomedicine, Chinese Academy of Sciences (GIBH, CAS, Guangzhou, China), the patients signed Informed Consent for donating their urine for stem cell research. The methods used in the study were carried out in accordance with stem cell research guidelines and regulations of GIBH, CAS. All experimental protocols and stem cell lines were approved by the Ethics Committee of GIBH, CAS.

## 3. Results

### 3.1. MEN1-iPSCs Obtained More Insulin-Positive Cells and Increased Insulin Production

Two MEN1 patients (SON and MOTHER) carrying the same mutation in exon 9 (c.1288GNT) of the MEN1 gene were characterized by PNETs [42]. Previously, we collected urine cells from those two patients and reprogrammed these cells into iPSCs with episomal plasmids (MEN1-iPSCs of the SON and MOTHER respectively) [36,37] and randomly selected an iPSC from a normal person without *MEN1* mutation as wild-type control [38].

To further investigate the pathogenesis of PNETs and hypoglycemia, MEN1-iPSCs were differentiated into insulin-producing cells (IPCs) through definitive endoderm cells (DECs) and pancreatic progenitor cells (PPCs) stepwise (Figure S1A). The stemness of those MEN1-iPSCs was similar to the WT-iPSCs and with similar DECs differentiation efficiency of WT-iPSCs (Figure S1B,C).

In the PPC stage, over 90% of the MEN1-PPCs were PDX1-positive and slightly higher than those driven from WT-iPSCs (about 80%) but without significance (Figure S1D). In the IPC stage, the percentage of insulin-positive cells for WT-IPCs, SON-IPCs, and MOTHER-IPCs was approximately 20%, 33%, and 40%, respectively (Figure 1A, the other clones are shown in Figure S2), corresponding to the insulin secretion ratio of approximately 1:2:4 (Figure 1B), and it was confirmed by the measurements of secreted C-peptides which presented a similar trend (Figure 1C). Moreover, the expression of NKX6.1, the specific marker of β-cells, was also increased in MEN1-IPCs (Figure 1D). These results demonstrated that an increased number of insulin-positive cells from MEN1-iPSCs resulted in increased total insulin production and secretion.

### 3.2. MEN1-Derived Cells Presented Neoplastic Features with Stepped up Proliferation Ability

Hyperplastic islets induced by the exceptional proliferative ability of β-cells are one major cause of hyperinsulinemia [43]. Cell cycle analysis at the PPC stage revealed that there was more replicative S phase status in MEN1-derived cells (Figure 2A and  Figure S3A). The cyclin proteins (B_1_, D_1_) were increased in MEN1-derived cells in PPC and IPC stage (Figure 2B). Furthermore, this result was also confirmed by proliferative analysis (Figure 2C and Figure S3B). Double immune-staining of Ki67 and the key pancreatic marker PDX1 at PPC stage found that the percentage of Ki67/PDX1 double-positive cells was higher in MEN1-derived cells, and the enhanced cell proliferation was observed from the PPC to the IPC stage (Figure 2D). However, proliferative abilities at the mature stage (Day 21) had no significant difference in MEN1 and WT cell lines (Figure S3C). These results indicated that MEN1-iPSCs modeled the phenotypes of MEN1 including the increased number of insulin-positive cells with enhanced proliferative abilities.

### 3.3. MEN1-Derived Cells Presented Increased GLP-1 Production and PI3K/AKT Signaling

The convertase PC1/3 existed in both α- and β-cells for GLP-1 and insulin maturation, respectively [21]. It was found that the PC1/3 level was significantly increased in MEN1-derived cells from the PPC to IPC stages (Figure S4A) and correlated with the increased GLP-1 expression and secretion (Figure 3A,B). In order to distinguish the cell type expressed PC1/3 in the IPC stage, the IPCs were sorted by a β-cells specific marker, CD49a^+^ [44], and we found that the expression of PC1/3 was upregulated in both CD49a^+^ (β-like cells) and CD49a^−^ (non-β endocrine cells) cells (Figure 3C). Furthermore, an increased PC1/3 was observed colocalized with glucagon in the MEN1-IPC stage (Figure 3D) resulting in more secreted GLP-1.

**Figure 4:**
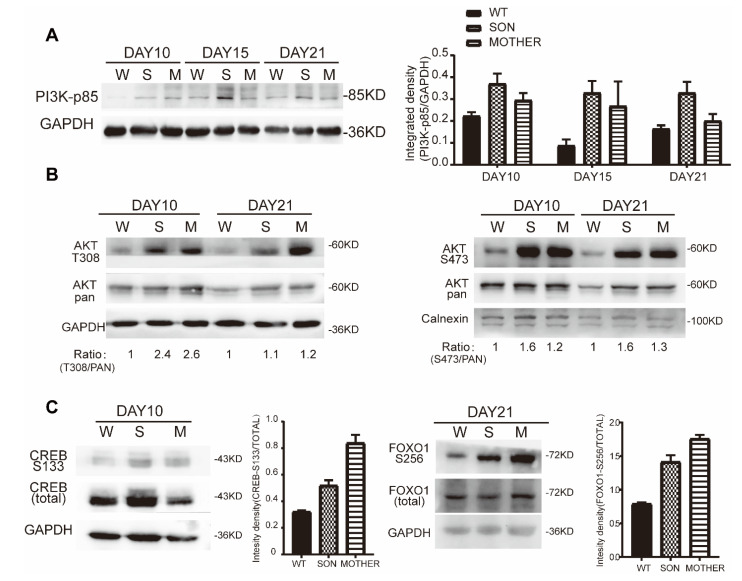
The increased expression of GLP-1R was accompanied by increased phosphorylation levels of key factors in the PI3K/AKT pathway in MEN1-derived cells. (**A**) The protein expression of PI3K-p85 between cell lines from PPC to IPC stage, *n* = 3; (**B**) *p*-AKT S473 and T308 phosphorylation was induced in MEN1-derived cells from PPC to IPC stage, respectively, *n* = 3; (**C**) AKT downstream transcription factor FOXO1 and CREB expression, *n* = 3; W-WT, S-SON, M-MOTHER.

Further, the expression of GLP-1R was confirmed in the PPC and IPC stage, mainly increased in CD49a^+^ β-like cells (Figure 3E). Since GLP-1R was mainly expressed in β-like cells, it was worth investigating whether GLP-1R-mediated proliferative signaling, PI3K/AKT, was activated. The regulatory subunit of PI3K, p85 was upregulated in MEN1-PPCs to IPCs (Figure 4A), leading to increased phosphorylation of S473 and T308 on AKT (Figure 4B).

Moreover, two AKT-regulated phosphorylation of *p*-CREB-S133 (Day 10) and *p*-FOXO1-S256 (Day 21) were consequently increased (Figure 4C), resulting in increased proliferative activity in the IPC stage. In summary, MEN1-derived cells represented increased production of GLP-1 and GLP-1R to facilitate PI3K/AKT-CREB/FOXO1 signal transduction to upregulate cell proliferation.

### 3.4. Inhibition of GLP-1R Signaling Rescued the MEN1-IPCs Phenotypes

**Figure 5:**
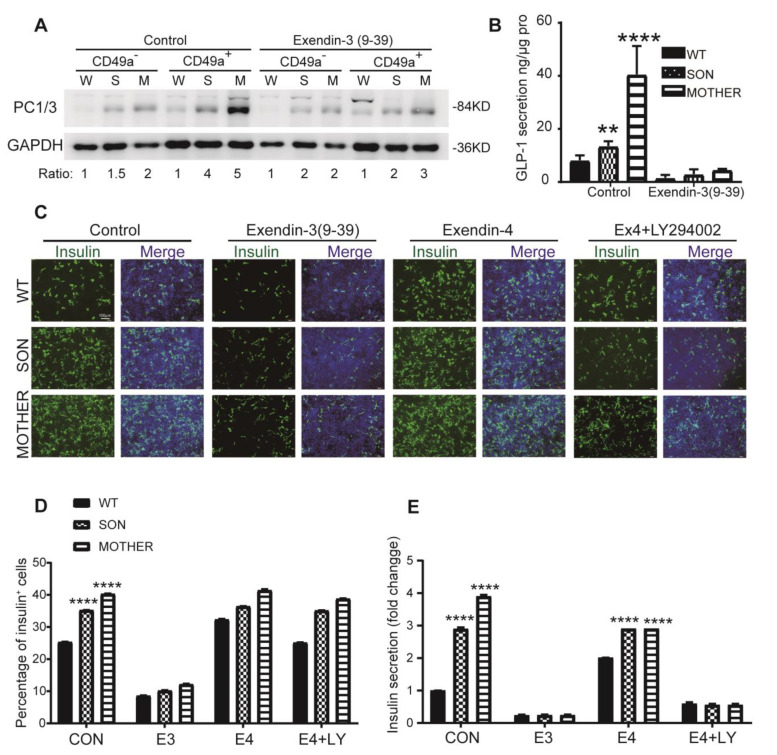
Inhibition of the GLP-1R with GLP-1 competitive ligand, Exendin-3 (9-39) inhibited the increased number of insulin-positive cells in MEN1-derived cells. (**A**) The protein expression of PC1/3 after cell-sorting with CD49a in IPC stage with Exendin-3 (9-39), *n* = 3. (**B**) ELISA measurement of GLP-1 in IPC stage with Exendin-3 (9-39), *n* = 3. (**C**) The immunostaining of insulin in IPC stage with Exendin-3 (9-39), Exendin-4 and Exendin-4 + LY294002, respectively, scale bar: 100μm and the quantitative results are shown in (**D**), *n* = 3. (**E**) ELISA measurement of insulin secretion in IPC stage, *n* = 3. W-WT, S-SON, M-MOTHER; CON-control, E3-Exendin-3 (9-39), E4-Exendin--4, LY-LY294002. The bar plots (*n* ≥ 3 wells per group) are mean ± SEM. ** *p* < 0.01 and **** *p* < 0.0001.

Our data suggested that upregulated GLP-1 expression might partly account for the increasing number of insulin-positive cells for MEN1-IPCs. In order to test whether blocking GLP-1 signaling could rescue the hyperproliferation and insulin hypersecretion, the competitive ligand of GLP-1, Exendin-3 (9-39), was administrated at the beginning of the IPC stage, and we monitored the cell proliferation characteristics. Upon the treatment, the expression of PC1/3 was reduced in MEN1-IPCs compared to WT-IPCs in both CD49a^+^ and CD49a^−^ cells (Figure 5A), a similar trend of change was observed for GLP-1 secretion (Figure 5B). These observations indicated that Exendin-3 (9-39) not only blocked the function of GLP-1, but also reduced its expression.

Furthermore, to verify whether GLP-1R activation was responsible for the phenotype of insulin hypersecretion, GLP-1R antagonist (Exendin-3 (9-39)), agonist (Exendin-4), and an inhibitor of PI3K (LY294002) were applied.

Under the Exendin-3 (9-39) treatment, the insulin-positive percentage in MEN1-IPCs decreased approximately to 15% at the similar level of WT-IPCs (a 3-fold reduction compared to control group), and it was also confirmed with the ELISA measurements of secreted insulin (Figure 5C–E); moreover, the higher level of Cyclin B1 in MEN1-derived cells (about 4-fold higher than WT-cells) was decreased to the same level of that in WT-cells (Figure S5A).

On the other hand, as expected, the GLP-1R agonist (Exendin-4) administration increased the number of insulin-positive cells, insulin secretion, and Cyclin B_1_ level in WT and MEN1-derived cells; however, the fold changes in MEN1-derived cells were much lower than in the WT-cells, since the GLP-1R signaling was already activated under MEN1 condition. Furthermore, the combination of Exendin-4 and LY294002 administration blocked the GLP-1R-agonist-induced insulin hypersecretion (Figure 5E). It suggested that PI3K pathway was downstream of GLP-1R.

### 3.5. Transplantation of MEN1-IPCs into NSI Mice Partially Recapitulated some Features of the Original Tumor in Patient

**Figure 6:**
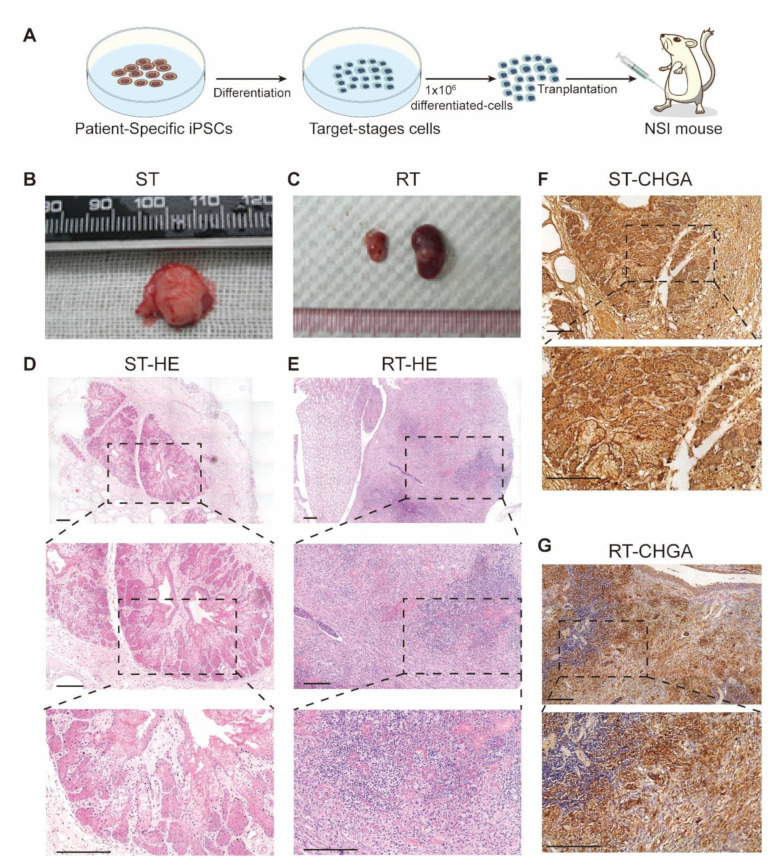
Transplantation of MEN-IPCs into NSI mice generated tumor similar as the original tumor from the patient. (**A**) The illustration of cell transplantation process. (**B**) The photograph of the tumor generation from SON-IPCs after 76 days of subcutaneous transplantation, marked as ST. (**C**) The tumors produced from subrenal capsular transplantation of SON-IPCs after 56 days, marked as RT. Hematoxylin and eosin staining for ST (**D**) and RT (**E**). (**F**) and (**G**) Immunohistochemical analysis of CHGA (PNETs biomarker) on tumors generated after transplantations; Scale bar, 200 μm.

To test whether MEN1-derived cells still hold tumorigenesis potential, different stages of MEN1-derived cells were transplanted into immune-deficient NSI mice (Figure 6A). There were no tumors generated from either the WT-derived cells or the MEN1-derived cells in the DEC or the PPC stage. However, when the SON-IPCs were transplanted subcutaneously or in renal capsule, one mouse in each group gave rise to a tumor (10%). For the tumor from subcutaneous transplantation (marked as ST), the node was noticed 28 days after transplantation, and the tumor was harvested 76 days after transplantation (Figure 6B,D). Immunohistochemical analyses showed strong positivity of chromogranin A (CHGA), a pathological marker of MEN1 (Figure 6F). The other tumor observed from transplantation in the renal capsule (marker as RT) showed infiltrated cells into normal mice kidney tissue and the mouse was sacrificed 56 days after transplantation (Figure 6C,E), which also expressed CHGA (Figure 6G). Those tumors recapitulated the features of the original tumor from the patient [42]. Therefore, the tumorigenesis ability of those cells driven from the patient iPSCs was confirmed specifically in the IPC stage. Moreover, when the status of GLP-1 was examined, it revealed nearly 80% GLP-1 strong positive-cells in the patient original tumor, and this phenomenon was recapitulated in the MEN1-iPSC modeled tumor on NSI mice (Figure S6).

## 4. Discussion

With the establishment of *MEN1* knockout mice and cell lines, much progress has been achieved in understanding its molecular pathogenesis. However, MEN1 is a complicated inherited disease in terms of genotype–phenotype relationship, even when an identical human *MEN1* mutation was recaptured in a mouse model, whether it produces tumors and the tumor type were unpredictable [28,29]. The MEN1 patient-origin iPSCs presumably preserve the original genomic identity and the epigenetic memory. Therefore, it may be an ideal resource for more closely modeling MEN1 patient-specific phenotypes. Indeed, it has been proven that iPSC-based cancer modeling holds advances in pathological mechanism elucidation and biomarkers discovery [45,46,47,48].

In our previous report, a 23-year-old male MEN1 patient was hospitalized for hyperinsulinemia-induced morning hypoglycemic comas. CT scans revealed a pancreatic tumor (3.4 × 3.4 cm^2^). The mother of this patient was also found with a large tumor at the pancreatic head (10 × 9 cm^2^). Immunochemical analyses indicated that both tumors strongly expressed chromogranin A. Both patients were confirmed to carry the same *MEN1* heterogenous mutation [42].

Using iPSC lines derived from those two patients, we performed an in vitro stepwise differentiation process and found that the MEN1-derived cells had higher proliferative abilities, leading to increased insulin-positive cells and total insulin production. These behaviors closely recaptured the hyperinsulinemia symptoms. When we transplanted those MEN1-IPCs into the immune-deficient NSI mice, they also mimicked the tumorigenesis ability, generating tumors exhibiting critical pathological biomarkers observed in the original tumor from the patients. The observed cell stage/type-specific pathogenesis is not just a novel scenario for PNETs, but also provides an ideal model for mechanistic studies including cell–cell interaction and influence of microenvironment.

**Figure 7:**
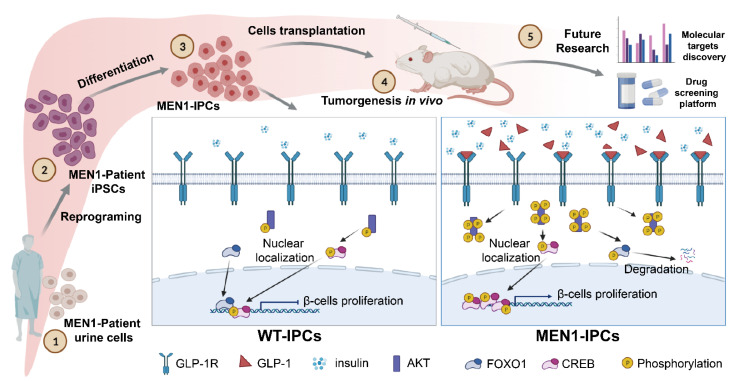
The illustration of MEN1 disease modeling with patient-origin iPSCs revealed GLP-1R-mediated hyperproliferation of β-cells and hypersecretion of insulin.

Based on MEN1-iPSCs modeling, mechanistically, we found that the hyperproliferation and insulin hypersecretion were GLP-1R dependent. The hyperproliferation was initiated in the PPC stage followed by increased expression of PC1/3 and GLP-1, leading to specific activation of GLP-1R on pre-β/β-like cells. The activation of GLP-1R resulted in PI3K/AKT-mediated sequential phosphorylation signaling transduction, disturbing the antagonistic balance between CREB and FOXO1. Consequently, more insulin-positive cells were produced in MEN1-derived cells. Blockages of either GLP-1R or PI3K significantly reduced the number of insulin-positive cells and the insulin hypersecretion (Figure 7).

It is interesting that MEN1 phenotypes appear in a temporal–spatial manner, and mechanisms should be related to the epigenetic memory. Indeed, menin participates in chromatin remodeling by partnering with MLL family proteins or Ezh2 to regulate methylation of histone H3 and cell proliferation [49,50,51]. The DNA methylation profile of PNETs of MEN1 was also strongly correlated with clinic outcomes [52]. Epigenetic changes (including DNA methylation and miRNAs expression) were relative to the aggression of MEN1 [53,54,55,56]. Therapeutic strategies to modify epigenetic status are in the developing process [57]. It is important to dissect the epigenetic alterations on the whole genomic level and our MEN1-iPSCs ideally meet this requirement.

In addition to the epigenetic profiling, the combination of omics analyses with transcriptome, proteome, and metabolome will help to identify key factors and their network for *MEN1* pathology. In particular, correlative analyses of proteome and metabolome will give clues for whether metabolic profile changes correlate with the change of protein expression. Those potential key targets will be validated by gene knockout or inducible overexpression. These studies will enable discovery of novel targets for diagnosis and drug development.

Patients with advanced and progressive PNETs have a poor prognosis and surgery is the primary option [58,59]. The limitation of effective treatment for this highly lethal disease raises the urgent need to develop new drugs for combination therapies. Ongoing drug development covers anti-proliferation and anti-secretion, epigenetic modifiers, and antagonists for WNT and VEGF signaling [60,61,62]. Now, we believe that the menin-associated signaling pathway should be considered as MEN1 drug target (s).

We found that in MEN1-derived cells, GLP-1 production was increased and it was also confirmed in the orthotopic tumor of the patient. The GLP-1R was specifically expressed in β-like cells. Inhibition of GLP-1R effectively reversed some phenotypes of MEN1, therefore, GLP-1R should be an ideal target. Furthermore, the GLP-1R agonists were already proven safe in clinical treatment for diabetes and obesity, it is worthwhile to develop antagonists of GLP-1R for PNETs treatment.

The percentages of pancreatic endocrine cells change upon aging and health conditions [63], whether *MEN1* affects pancreatic endocrine cell proportions remains unknown, and whether differences in the composition of its endocrine cells contribute to differences in the onset phenotype of MEN1 remains unknown. Given that in vitro differentiation protocols have certain limitations, we did not discriminate β-cells and α-cells as well as their endocrine progenitors well. This is due to the fact that the susceptibility of human iPSCs to differentiate into cells with an immature characteristic rather than a fully mature adult state [64]. Those questions are worth being validated by collecting a larger number of patient samples and continuing improvement of the MEN1-iPSC model.

In conclusion, MEN1 patient-derived iPSCs not only closely mimicked some of the phenotypic features of PNETs *in vitro*, but also maintained the tumorigenesis potential. Furthermore, our MEN1-iPSC lines will serve as a platform for MEN1 drug discovery by focusing on the manipulation of GLP-1R signaling.

## Supplementary Materials

The following supporting information can be downloaded at: https://www.mdpi.com/article/10.3390/cells11152387/s1. Table S1. Antibodies used in the study; Figure S1. MEN1-iPSCs had the same pluripotency in stem cell stage and similar differentiation efficiency in DEC stage; Figure S2. Significant increased insulin production of MEN1-IPCs from two more iPSC lines from each patient compared with two more wild type lines; Figure S3. Increased proliferative rate in MEN1-derived cells; Figure S4. Upregulation of GLP-1 signaling in MEN1-derived cells; Figure S5. Exendin-3 (9-39) inhibited the MEN1-derived cells into β-cells via inhibition on proliferation; Figure S6. The expression of GLP-1 was strong in MEN1-relative tumors.
