# Supplementary material for: Modeling MEN1 with Patient-Origin iPSCs Reveals GLP-1R Mediated Hypersecretion of Insulin
Source: Cells. 2022 Aug 3;11(15):2387. doi: 10.3390/cells11152387 (PMC9368616; doi:10.3390/cells11152387)
Supplement: Supplementary file 1 [file cells-11-02387-s001.zip › cells-1713328-supplementary.pdf]

cells MDPI

Article

Modeling MEN1 with Patient-Origin iPSCs Reveals GLP-1R Mediated Hypersecretion of Insulin

Supplementary Materials

Table S1. Antibodies used in the study.

Primary	Source	Cat. No.
Goat Anti-Sox17	R&D	AF1924
Rabbit Anti-FoxA2	Genetex	GTX84485
Goat Anti-Pdx1	R&D	AF2419
Rabbit Anti-SOX9	Abcam	AB185230
Mouse Anti-Glucagon	Abcam	R0539L-1
Rabbit Anti-Insulin	Cell Signaling Technology	3014
Mouse Anti-Ki67	GeneTex	MKi67/2462
Rabbit Anti-FOXO1	Cell Signaling Technology	2880
Rabbit Anti-*p*FOXO1	Cell Signaling Technology	9461
Rabbit Anti-CREB	Cell Signaling Technology	9197
Rabbit Anti-*p*CREB	Cell Signaling Technology	9198
Rabbit Anti-AKT-pan	Cell Signaling Technology	4691
Rabbit Anti-AKT-S473	Cell Signaling Technology	4060
Rabbit Anti-AKT-T308	Cell Signaling Technology	13038
Rabbit Anti-OCT4	GeneTex	GTX101497
Rabbit Anti-Nanog	Cell Signaling Technology	4903S
Rabbit Anti-GLP-1R	Proteintech	26196-1-AP
Mouse Anti-PCNA	Abcam	ab29
Rabbit Anti-Chromogranin A	Abcam	ab254557
Rabbit Anti-GLP-1	Abcam	ab22625
Rabbit Anti-PCSK1(PC1/3)	Boster	BA2848
PE Mouse Anti-Human CD49a	BD Pharmingen	V S223
Rabbit Anti-NKX6.1	Cell Signaling Technology	54551S
HRP-conjugated Beta Tubulin	Proteintech	HRP-66240
HRP-conjugated Beta Actin	Proteintech	HRP-60008
HRP-conjugated Gapdh	Proteintech	HRP-60004
Donkey Anti-Rabbit 568	Life	A21206
Donkey Anti-Goat 488	Life	A11057
Donkey Anti-Mouse 488	Life	A21202
Donkey Anti-Mouse 568	Life	A10037
Donkey Anti-Rabbit 488	Life	A21206
Goat Anti-Rabbit HRP	Kangcheng	KC-RB-035
Goat Anti-Mouse HRP	Kangcheng	KC-MM-035

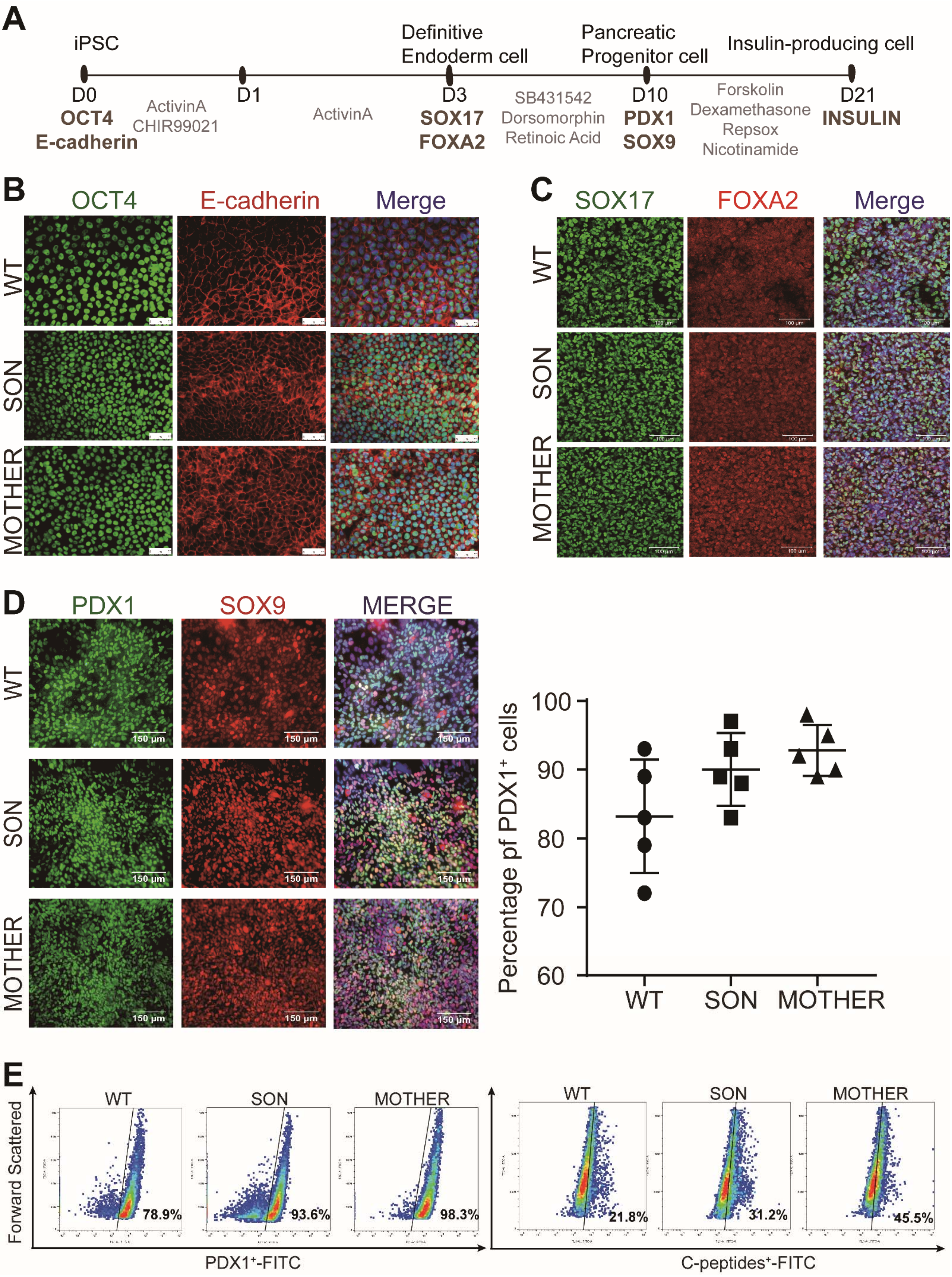
A
iPSC
Definitive Endoderm cell
Pancreatic Progenitor cell
Insulin-producing cell
D0
OCT4
E-cadherin
ActivinA
CHIR99021
D1
ActivinA
D3
SOX17
FOXA2
SB431542
Dorsomorphin
Retinoic Acid
D10
PDX1
SOX9
Forskolin
Dexamethasone
Repsox
Nicotinamide
D21
INSULIN
B
OCT4
E-cadherin
Merge
WT
SON
MOTHER
C
SOX17
FOXA2
Merge
WT
SON
MOTHER
D
PDX1
SOX9
MERGE
WT
SON
MOTHER
150 μm
Percentage pf PDX1+ cells
100
90
80
70
60
WT
SON
MOTHER
E
Forward Scattered
WT
SON
MOTHER
78.9%
93.6%
98.3%
PDX1+-FITC
WT
SON
MOTHER
21.8%
31.2%
45.5%
C-peptides+-FITC

Figure S1. MEN1-iPSCs had the same pluripotency in stem cell stage and similar differentiation efficiency in DEC stage, scale bar: 50μm; (**A**) The stepwise pancreatic differentiation protocol of iPSCs is illustrated. (**B**) The pluripotency of iPSCs was analyzed by E-cadherin and OCT4 immunostaining, scale bar: 100μm. (**C**) The definitive endoderm cell stage was identified by FOXA2 and SOX17 immunostaining, scale bar: 150μm. (**D**) The pancreatic progenitor stage (Day 10) was identified by PDX1/SOX9 immunostaining, $n = 5$. (**E**) Analysis of PDX1 in Day 10 and insulin in Day 21 by flow cytometer. The bar plots ($n \geq 3$ wells per group) are mean ± SEM.

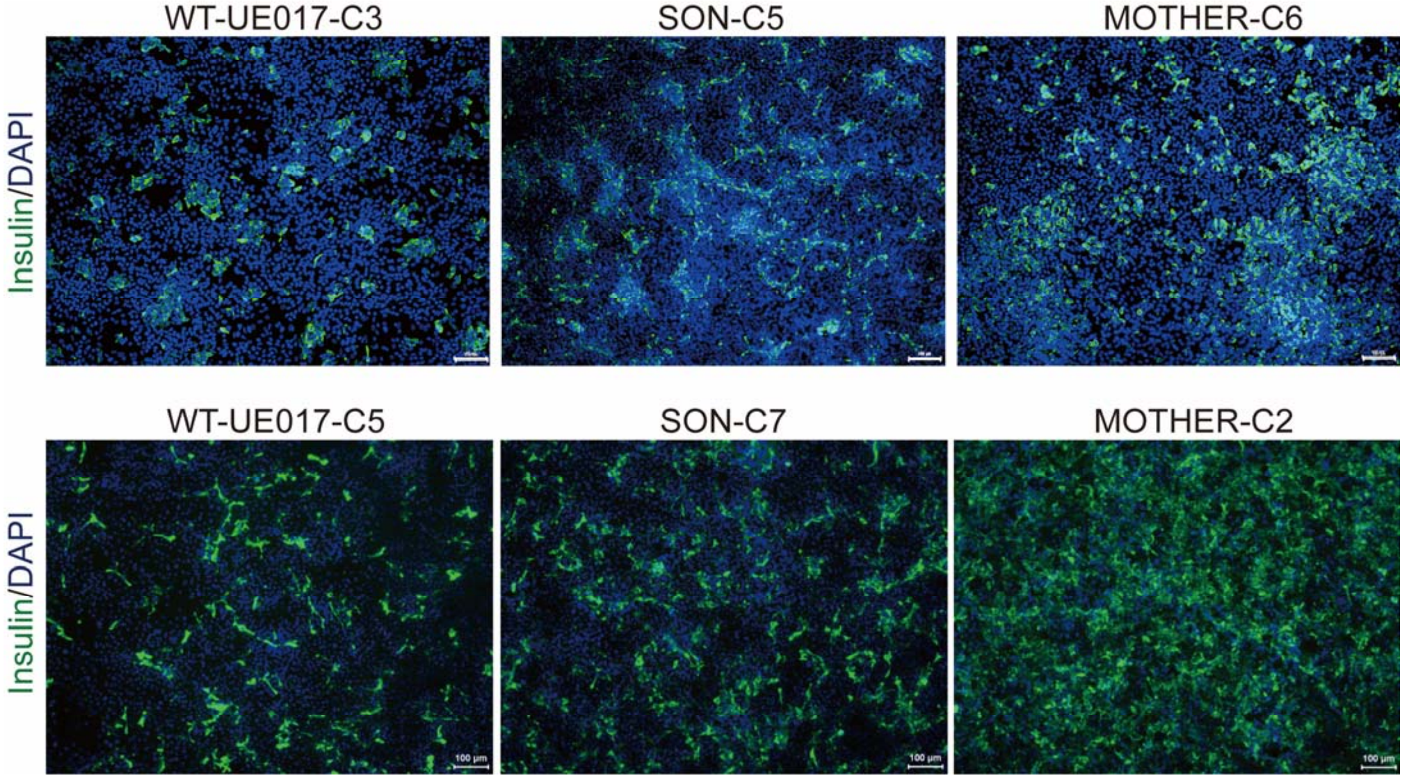

Figure S2. Significant increased insulin production of MEN1-IPCs from two more iPSC lines from each patient compared with two more wild type lines, scale bar: 100μm.

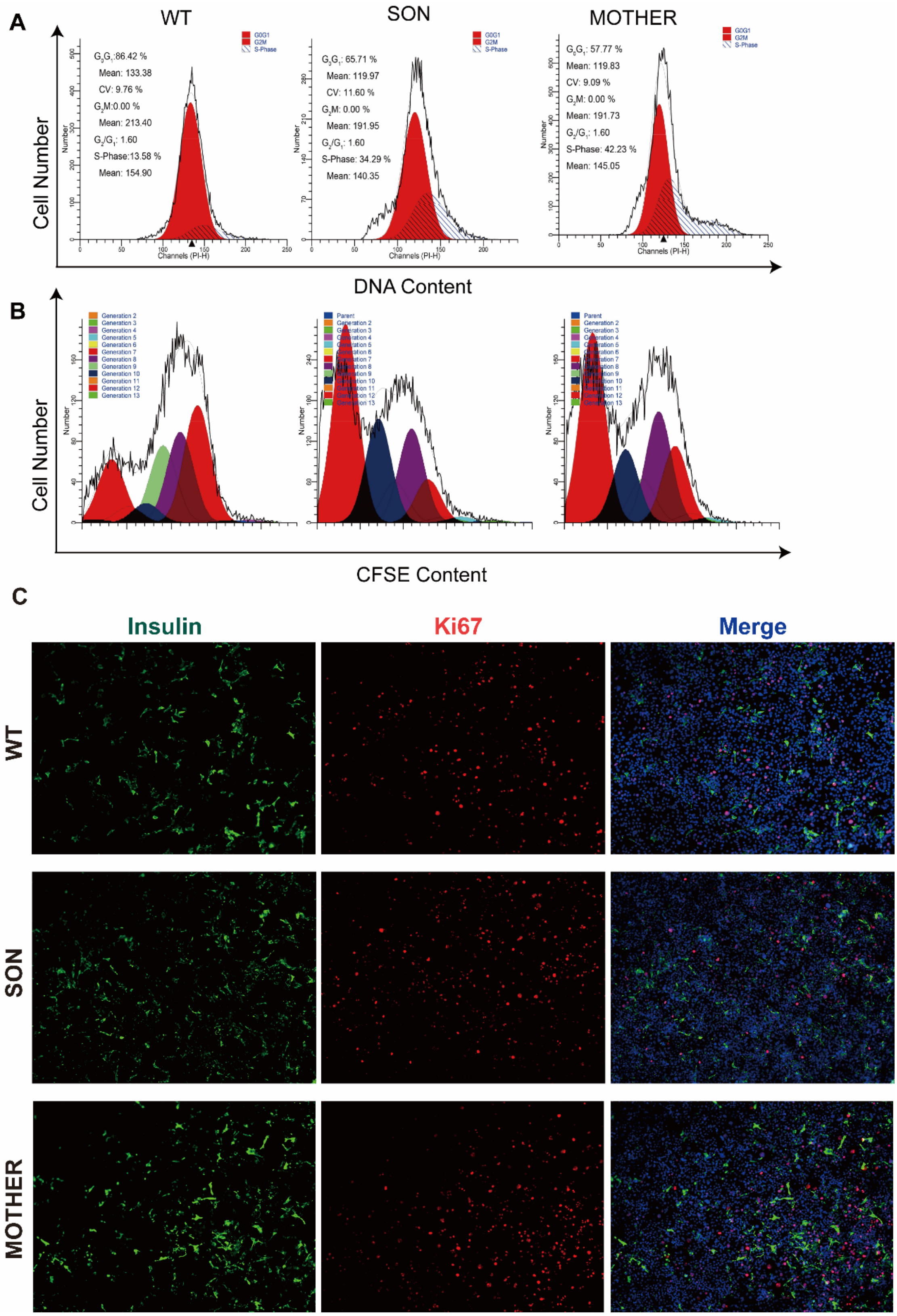

Figure S3. Increased proliferative rate in MEN1-derived cells. (**A**) Flow cytometer analysis of DNA content to identify cell cycle by PI in Day 10. (**B**) Flow cytometer analysis of cell proliferation by CSFE from Day 7–15. (**C**) The proliferation of IPC stage was identified by Ki67 and insulin.

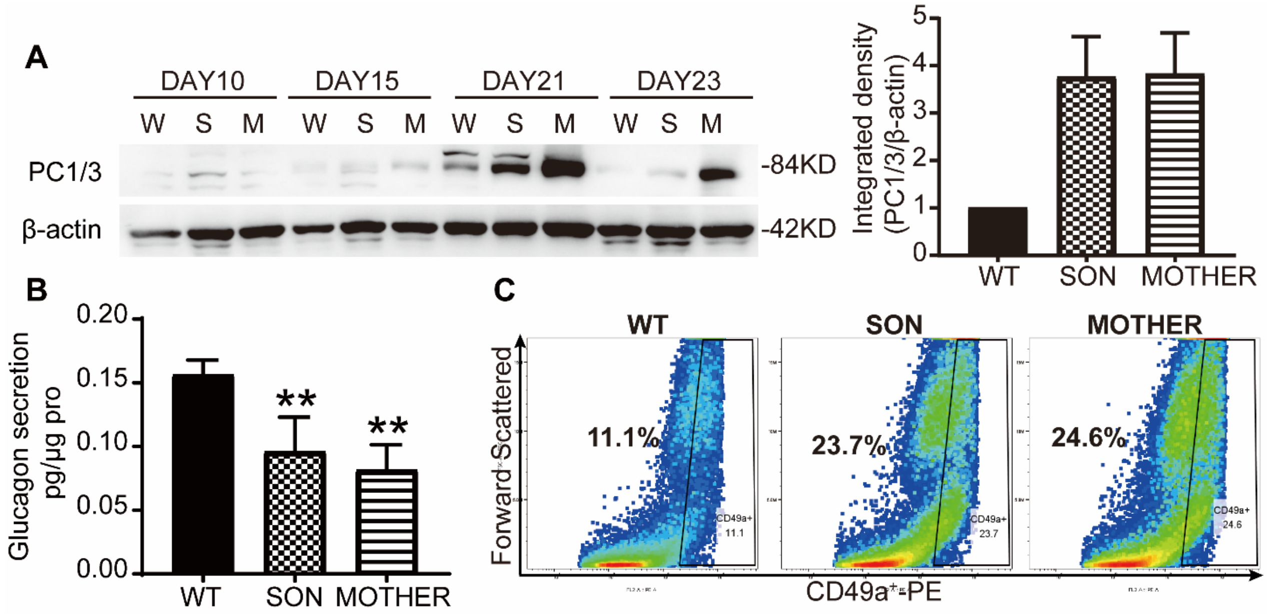

Figure S4. Upregulation of GLP-1 signaling in MEN1-derived cells. (**A**) The expression of PC1/3 during the differentiation process (n = 3). (**B**) ELISA assay of glucagon secretion in supernatant of Day 21 (n = 3). (**C**) Cell-sorting by CD49a$^+$ in Day 21 by flow cytometer. The bar plots ($n \geq 3$ wells per group) are mean ± SEM.

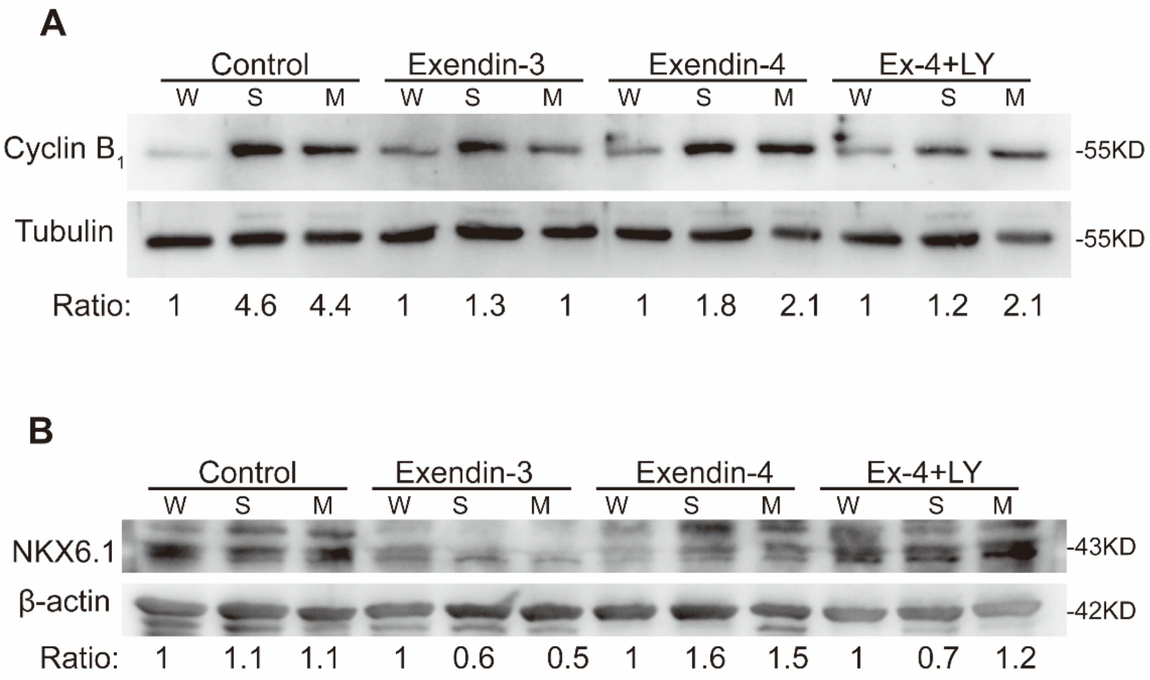

Figure S5. Exendin-3 (9-39) inhibited the MEN1-derived cells into β-cells via inhibition on proliferation. (**A**) The expression of Cyclin B1 in IPCs treated with Exendin-3 (9-39), Exendin-4, or Exendin-4 + LY294002. (**B**) The expression of β-cells specific marker NKX6.1 in IPC stage treated with Exendin-3 (9-39), Exendin-4, or Exendin-4 + LY294002.

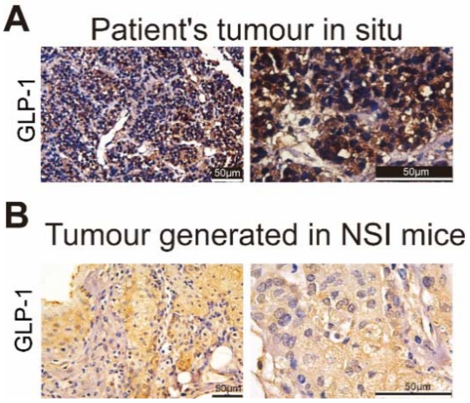

Figure S6. The expression of GLP-1 was strong in MEN1-relative tumors. The immunostaining of GLP-1 from patient tumor of SON sample (**A**) and tumor generated in NSI mice after SON-IPCs transplantation (**B**), scale bar: 50μm.
